# Enhanced Skin Disease Classification via Dataset Refinement and Attention-Based Vision Approach

**DOI:** 10.3390/bioengineering12030275

**Published:** 2025-03-11

**Authors:** Muhammad Nouman Noor, Farah Haneef, Imran Ashraf, Muhammad Masud

**Affiliations:** 1 School of Computing, National University of Computer & Emerging Sciences (FAST-NUCES), Islamabad 44000, Pakistan; 2 Department of Software Engineering, Capital University of Science and Technology (CUST), Islamabad 44000, Pakistan; 3 Department of Electrical Engineering, College of Engineering, University of Business and Technology, Jeddah 21361, Saudi Arabia

**Keywords:** skin disease, preprocessing, classification, vision transformer, skin cancer

## Abstract

Skin diseases are listed among the most frequently encountered diseases. Skin diseases such as eczema, melanoma, and others necessitate early diagnosis to avoid further complications. This study aims to enhance the diagnosis of skin disease by utilizing advanced image processing techniques and an attention-based vision approach to support dermatologists in solving classification problems. Initially, the image is being passed through various processing steps to enhance the quality of the dataset. These steps are adaptive histogram equalization, binary cross-entropy with implicit averaging, gamma correction, and contrast stretching. Afterwards, enhanced images are passed through the attention-based approach for performing classification which is based on the encoder part of the transformers and multi-head attention. Extensive experimentation is performed to collect the various results on two publicly available datasets to show the robustness of the proposed approach. The evaluation of the proposed approach on two publicly available datasets shows competitive results as compared to a state-of-the-art approach.

## 1. Introduction

Addressing the fact that the skin is the largest organ in the human body, dermatological diseases are listed among the most frequently encountered illnesses [1]. Some of them are evident and do not pose a terrible threat when treated, while other ones are critical and can be life-threatening if not treated in time. In fact, when they are diagnosed at the terminal stages, approximately 30% to 70% of individuals belong to high-risk groups [2]. Currently, there are estimated to be approximately 2 to 3 million new cases of non-melanoma skin cancers, as well as approximately 132,000 new cases of melanoma skin cancers annually [3]. Malignant melanoma is one of the most dangerous forms of the disease that leads to 10,000 mortalities per year all over the world [4]. Early detection of any abnormality in the melanocytes has a high rate of survival of 96% when diagnosed in the early stages, as compared to 5% in the late stages [5]. The WHO has estimated that, on average, death occurs to the tune of forty per one hundred thousand population with skin diseases, according to epidemiological findings [6]. Due to the thinning of the ozone layer, and its acting as a shield to the sun rays, the increased radiation with UV-B and UV-C has become more common on the surface of the earth. Therefore, this higher UV exposure increases non-melanoma skin cancers that play a major role in the development of malignant melanoma [7]. Many AI-based systems have been developed to help doctors like OPD, OR systems, and advanced health data analysis systems like IBM Watson, Tempus, and Cerner for medical research, finding cancer treatments and electronic health records, respectively [8]. But doctors still rely on their ability to diagnose the disease by drawing their attention to visually detected symptoms such as color and scaling of the lesions as well as their distribution.

Lack of awareness and over-optimistic attitudes cause people to think their skin diseases are not severe, and so they try home remedies [9], potentially worsening the condition [10]. Since skin diseases are easily communicable, it becomes important that they are treated in their early stages [11]. As a result, there is a clear need to solve this problem and guarantee the appropriate and timely treatment of skin diseases at the initial stage to avoid further damage. Furthermore, despite the fact that existing systems are impressive in terms of accuracy, they show certain restrictions if the number of diseases, which can be detected in one analysis, is concerned. These systems often focus on a comparatively narrow number of diseases, and this is typically not more than three or five diseases in most cases.

Despite promising advancements, some issues and challenges remain with regard to AI-based skin disease diagnosis. One major challenge is that some skin diseases like skin cancer and vitiligo manifest minimal pathological symptoms in their early stages [12,13,14]. Many conventional diagnostic techniques used in dermatology are based on observation and gross examination, resulting in a lack of well-defined standards, measures, and quantity, which can potentially lead to misdiagnosis, even by dermatologists with many years of experience [15,16]. The lack of dermatologists in remote areas means that other clinicians, who may not have adequate understanding and skills in the diagnosis and management of dermatological diseases, conduct these procedures, thus worsening the possibility of incorrect diagnosis. This is compounded by the fact that there is an inequality in the distribution of healthcare facilities, thus it becomes hard to make an accurate diagnosis.

Despite the potential of AI technology, especially image recognition [17,18,19,20,21], several drawbacks should be noted. For instance, patterns used in the AI models have to be carefully coded as well as tested to eliminate bias when making diagnoses. Both ML and DL can effectively recognize and outline similar characteristics of skin lesions and therefore lead to the correct identification of the moles; however, their efficiency depends on the volume and heterogeneity of the data set. DL algorithms tend to be more effective when a large dataset is available while ML is effective when the data volume is small [22].

The main contributions of this research are as follows:

- Utilization of advanced image processing techniques to improve the quality of the images. Quality of the images is evaluated using peak-signal-to-noise-ratio (PSNR) and mean squared error (MSE).
- Development of attention-based framework for efficient skin disease classification. The proposed framework utilizes the encoder part of transformers with multi-head attention for improved performance.
- Evaluation of proposed framework on various performance measures using two publicly available datasets.

The rest of the paper is organized as follows: The next section presents literature, afterwards methodology is explained, and subsequently results are presented. Finally, the conclusion of the paper is shown.

## 2. Related Literature

Skin disease recognition and classification have been investigated in detail with such approaches through different AI methods, which indicates promising incremental improvements in terms of accuracy and time. Chen et al. [23] proposed skin disease recognition through the closed loop learning model with wide data acquisition from itself. Their study employed large datasets processed using LeNet-5, AlexNet, and VGG16, which proved to be quite efficient in identifying skin conditions. Kawahara et al. [24] proposed an architecture of a multi-resolution-tract convolutional neural network (CNN) with both pretrained and skin-lesion-trained layers. The combination of analysis for skin texture classification yielded higher accuracy as compared to the existing techniques of machine learning, thereby underlining the benefits of MTM in improving the classification rate of hybrid models. Ismael et al. [25] addressed an advanced deep learning technique for the classification of brain cancer MRI images using residual network, and information is available as the utility of deep learning techniques for classification of various types of skin diseases such as meningiomas, gliomas, and pituitary tumors. This is a testimony of how DL models are flexible when performing analysis of hologram medical images. Garnavi et al. [26] focused on thermal, surface, and dermoscopy images and formed the basis of border detection for dermoscopy images using hybrid thresholding on optimized color channels, intending to offer further automated border detection to aid diagnostic accuracy. It marks that their work belongs to the earlier studies of dataset collections and improves the accuracy of skin disease prediction. Chieregato et al. [27] proposed the use of a combination of machine learning and deep learning in predicting COVID-19 severity based on CT scans and patients’ clinical information, proving that AI-based models could be used to predict the outcomes from medical imaging data. Hence, this research paper supports the possible use of AI in diagnosing skin diseases as well as managing them, in conformation with the general enhancements in AI-based healthcare solutions.

Based on classification, convolutional neural networks (CNNs) have been widely applied in dermostoscopic image analysis (DIA) since 2015. Recent studies of computer vision and digital image processing have highlighted the importance of deep learning procedures in attaining precision in segmentation, detection, and classification, especially for challenging tasks. Codella et al. [28] have demonstrated the usefulness of deep residual networks and CNN models to distinguish malignant lesions, therefore exhibiting high performance in dermatological applications. Hybrid deep neural networks have also indicated potential in improving the performance of the diagnosis. Thomas et al. [29] proposed a deep learning architecture for skin lesion segmentation and classification which includes categorizing the tissues into 12 dermatologist classes. When applied to dermoscopy images, this framework established a computer-generated accuracy of 97% compared to the clinical method, which was 93.6%. Implementing a deep learning model with the incorporation of other standard models has been shown to enhance the results in classification. Amin et al. [30] proposed an integration approach for deep feature fusion that makes use of preprocessing, segmentation, and feature-extracting methodologies. To increase the efficiency of the segmentation process, they resize images, convert RGB to the luminance channel, and use Otsu and Biorthogonal 2D wavelet transform, etc. To overcome these problems, the authors used deep feature extraction techniques from the pretrained models of AlexNet and VGG16 and applied principal component analysis (PCA) for feature selection and reduced the dimensionality of the features with good classification accuracy. Masni et al. [31] designed a deep learning architecture which assimilates all these phases, with FRCN for segmentation and various classifiers such as Inception-v3, ResNet-50, or Inception-ResNet-v2 for classification. The high accuracy of their method was evident in the ISIC2016, ISIC2017, and ISIC2018 datasets, and the authors rightly noted the importance of integrated deep-learning models in dermatological applications. In another study, El-Khatib et al. [32] used the ResNet-101 model for skin lesion classification on the PH2 database, as they used fine-tuned CNN models with transfer learning to detect various types of skin lesions. This approach came to around 90% accuracy, which shows how useful it is to use a pretrained model for skin disease detection.

## 3. Material and Methods

Initially, the dataset is collected, and subsequently the multiple preprocessing techniques like adaptive histogram equalization (AHI), binary cross-entropy with implicit averaging (BCEI), gamma correction, and contrast stretching are performed on the images. Subsequently, a deep-learning framework based on vision transformers (ViT) is applied to the preprocessed images. Finally, classification is performed.

**Figure 1:**
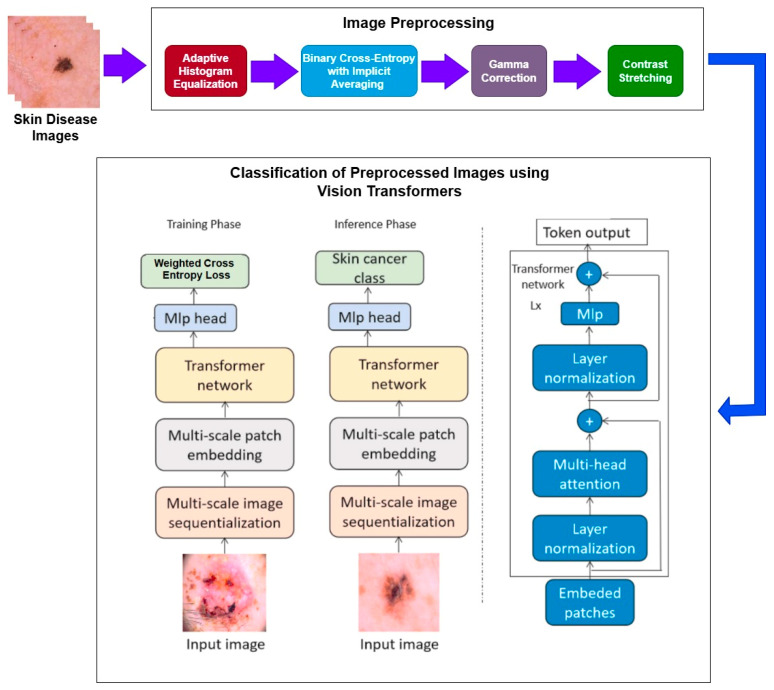
Methodology diagram.

The research encompasses a structured approach and brings out a sequence of specific procedures in analyzing images. The initial process comprises detailed image preprocessing, which is very important when it comes to enhancing the image quality and, hence, improving the accuracy. This is a very important step that includes removing noise from the images and resizing and normalizing the images and data for it to go through the various other analyses that are required. Lastly, as envisioned earlier, the vision transformer (ViT) classification model is used after the data preprocessing stage is completed. ViT is a novel paradigm shift in the analysis of images where the given transformer architectures operate on images as sequences of patches. This model is unique and notably so in terms of evaluating images from a perspective that isolates them through the identification of traits that make the images unique in relation to the rest of the images which aids in classifying the images. The steps followed for the given methodology are shown in Figure 1.

### 3.1. Dataset Description

This study employed two datasets: the skin disease image dataset [33] as well as HAM10000 [34] dataset, each serving specific purposes in classification in the domain of skin diseases. The dataset was dedicated to the classification problem and involved 10 significant dermatological diseases. Eczema, warts, molluscum and other viral infections, melanoma, atopic dermatitis, basal cell carcinoma, melanocytic nevi, benign keratosis-like lesions, psoriasis, lichen planus and related diseases, seborrheic keratoses, and tinea ringworm candidiasis are the skin diseases which differ by their intensity and importance.

These diseases are all important in the field of dermatology as well as the general healthcare field. Eczema, for instance, refers to a type of skin inflammation that is quite common and known to affect millions of people in different parts of the world, thus leading to a lot of discomfort and reduced quality of life. While basal cell carcinoma and squamous cell carcinoma are relatively non-threatening, melanoma is one of the most dangerous skin cancers, which underlines the problem of accurate classification to enhance diagnosis. Basal cell carcinoma is the most frequently occurring skin cancer and to some extent is less dangerous than melanoma; nevertheless, it is important for clinics and medical practices to be aware, as accurate diagnosis methods are needed. Furthermore, psoriasis and atopic dermatitis are types of skin diseases where patients have to live a long time with pain and a changed appearance of skin. Distribution of images across various skin disease classes are shown in Table 1.

The work relied on the HAM10000 dataset to establish skin diseases, which are rich in numbers and kinds of images. This set of images includes a vast array of dermatological pathologies necessary for realistic object detection. The diseases identified in the HAM10000 dataset are actinic keratosis clinical (AKIEC), basal cell carcinoma (BCC), benign keratosis (BKL), dermatofibroma (DF), melanoma (MEL), melanocytic vevi (MN), and vascular malformations (VASC).

**Figure 2:**
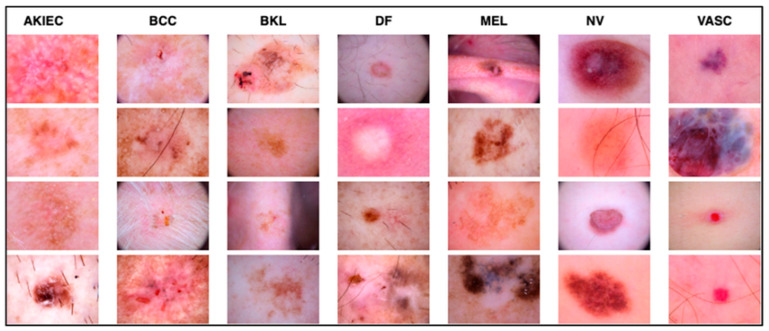
Sample images from HAM10000 dataset.

The diseases presented in the HAM10000 dataset are grouped into different categories, and each of them has its own challenges and things to consider in terms of diagnosis. The samples images from HAM10000 are shown in Figure 2. Actinic keratoses (AKIEC) are photocarcinomas resulting from acts of sun irradiation, which require identification from the early stages to prevent transition to carcinoma. Another study is needed on another type of frequent skin cancer, basal cell carcinoma (BCC), which has several subtypes with different clinical manifestations that demand correctly identifying algorithms. This article discovers that benign keratosis (BKL) and dermatofibroma (DF) are skin lesions that can mimic malignant skin lesions; therefore, a precise object detection method is crucial in differentiating benign and malignant skin lesions. As noted earlier, MEL is such a crucial object of detection concerns due to its invasive and metastasizing potential. Melanocytic nevi or common moles are benign growths but sometimes can very closely mimic melanoma and, therefore, needs more refined diagnostic approaches. Furthermore, vascular malformations (VASC) refer to common skin afflictions that must, therefore, be diagnosed correctly for appropriate treatment methods to be applied.

### 3.2. Image Preprocessing

The methodology employed in this study involved various techniques in the processing of image data so as to improve the quality and interpretability of skin disease images. It was possible to achieve that by choosing four techniques, the potential of which lies in enhancing the contrast, sharpness, and overall quality of the image. These techniques were selected for use in order to meet the task of correct picture recognition of various skin ailments while taking into account peculiarities and difficulties inherent in image dermatology.

The choice of these image processing techniques was guided by the fact that skin disease images required some preprocessing to enhance the features needed in the classification stage and object detection stages. The reason why adaptive histogram equalization has been chosen is the capacity to redistribute pixel intensities and the necessity to improve contrast in regional sections of images, which is essential when identifying various characteristics of skin lesions. Binary cross-entropy with implicit averaging (BCEI) was added as its enhanced version because of the inclusion of both brightness and contrast enhancement, making it a rounded method for normalizing the intensity values of images and enhancing the appearance of the images. Furthermore, the mechanism of gamma correction was added to define brightness and contrast so that visually appealing images that could be compatible with a variety of display devices that were possessed. Contrast stretching was the most appropriate to enhance the contrast and make images visually clear and interpretable; therefore, it has useful applications in medical diagnostics and research in skin diseases.

#### 3.2.1. Adaptive Histogram Equalization (AHE)

Adaptive histogram equalization that is also known as local histogram equalization aims at enhancing the contrast of an image with the help of redistributing pixel intensities. It uses the histogram equalization method and applies this to specific regions of an image so that the contrast adjustment is wise. This technique aims to improve the image resolution for improved computer usage for interpretation and analysis.

#### 3.2.2. Binary Cross-Entropy with Implicit Averaging (BCEI)

BCEI is another preprocessing technique that incorporates several brightness and contrast enhancement procedures to bring the intensity levels of an image to a similar range. It entails processes such as histogram regulation, and contrast expansion that help enhance the look of images. BCEI is also concerned with raising the standard of images to enable proper interpretation and analysis.

The implicit averaging of the data is calculated when we have noisy data in the image [35]. To denoise the image, implicit averaging [36] is utilized, and the improvement among the pixels is calculated using binary cross entropy to further optimize the noisy image. Binary cross entropy is measured using the given equation.

$$BCE=-\frac{1}{N}\;\sum_{i=1}^{N}\left(P_{orig}\;\cdot \;log(P_{New})+(1-P_{orig})\;\cdot \;log(1-P_{New})\right)$$

Here, $P_{orig}$ is the original pixel value, and $P_{New}$ represents the value after the implicit averaging, and N is the total number of pixels in the image.

#### 3.2.3. Gamma Correction

Gamma correction is a process of brightness and contrast enhancement as an image processing technique to optimize the appearance of images and their suitability for use on any display hardware. Converting pixel values enhances visibility and accurate depiction on screens, which is useful in computer vision, graphical interfaces, and imaging.

#### 3.2.4. Contrast Stretching

Contrast stretching is a preprocessing technique that increases the span of subtle strength settings in an image, making hidden areas more visible and giving a better picture quality. This technique has applications in computer vision and remote sensing as well as in the medical field.

### 3.3. Classification

**Figure 3:**
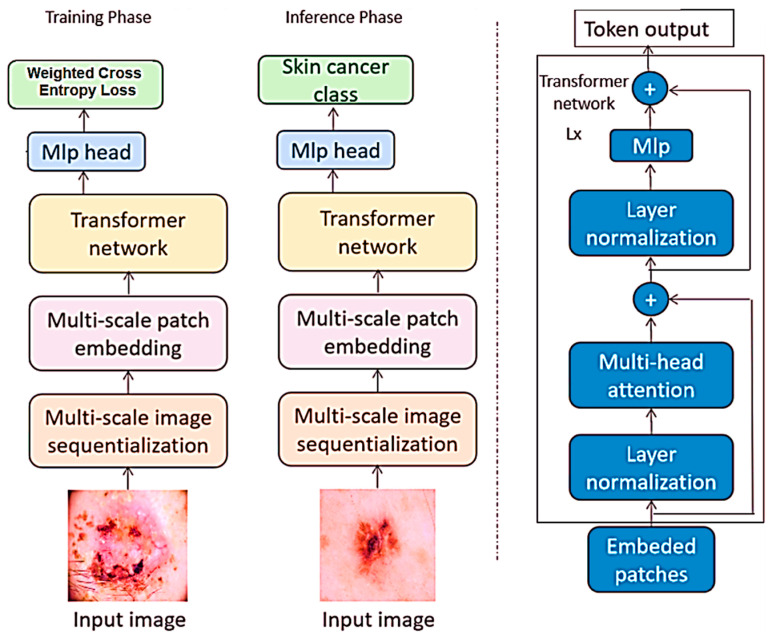
Framework of vision transformers.

In the realm of image classification, this study utilized vision transformers as the deep-learning model for analyzing images which are newly developed, especially in the field of image processing. The term vision transformers refers to the model that applies to the transformer networks, which are efficient in realizing sequential data to image classification problems. While conventional CNNs feed images as two-dimensional grids of pixels, vision transformers feed images as sequences of fixed-region patches. As for the differences between the discussed vision transformers and the classic CNN models, the latter take pixel grids of the images as input or feed, while the former works with patches. This capability allows the network to establish long-range connections within the image to improve the performance of various image classification tasks. The framework of vision transformers is shown in Figure 3.

The selection of vision transformers was informed by the results, proving the efficacy of the models in various image classification undertakings such as the ILSVRC or ImageNet. Also, it is important in object and scene analysis and therefore makes it a suitable algorithm for different forms of image classification. Moreover, several advantages can be derived from using vision transformers in comparison to the usual deep-learning frameworks; training them with limited data samples is quite possible, and it can accept input images of any dimension possible without the need to crop or resize the input image by hand options, which are quite common in practical applications.

In this work, the vision transformer model under study operates as it detects patches of images instead of CNN architectures. Each patch is flattened into a linear embedding, on which these learnable feature vectors are dropped through a transformer for prediction. The extracted patches are transformed and fed into the model by permutations without the loss of information, and, in the classification, the phase aids the model to capture the long-range dependency and context.

Unlike traditional computer vision models that work directly on images, in-vision transformer images are split into patches and then fed through the transformer architecture that incorporates attention layers, which opens up new possibilities of how images can be processed. Such architectural design starts with the subdivision of the input image into small regions called grid cells. In this approach, each patch is then represented as a vector. The transformation process involves several steps outlined along with equations.

#### 3.3.1. Patch Embeddings

Initially, the image is divided among the fixed size patches. In our case, the patch size was set to 16. The patch is then flattened into a 1D vector, which for an RGB image will result in a 768-dimensional vector. Every patch which is flattened is linearly embedded into the vector. Positional embeddings are further added with each patch.

Each patch xi is flattened into a vector ${x_{i}}^{E}$. These flattened patches are then projected through an embedding matrix F to produce a linear patch projection, which represents the projection dimension. This can be represented as


$$z_{0}=\left[I_{class};x_{1}^{E};x_{2}^{E};\ldots ;x_{n}^{E}+E_{pos}\right]$$

Here, $E_{pos}$ is the learnable class embedding, and $I_{class}$ represents the class tokens concatenated with the patch embeddings.

#### 3.3.2. Transformer Encoder

These patch embeddings are then passed into a model called ‘transformer encoder’, which contains as the key components what is also known as multi-headed self-attention (MSA) that consists of multiple linear self-attention/linear projection blocks and multi-layer perceptron (MLP) blocks. Self-attention could allow residual skip connections to be added in conduit through layer normalization (LN) after each transformer encoder block. The operations of MSA and MLP blocks can be expressed as expressions of the MSA and MLP block operations:


$$z_{l}^{\prime }=MSA\left(LN\left(z_{l-1}\right)+z_{l-1}\right)$$

$$z_{l}=MLP\left(LN\left(z_{l}^{\prime }\right)+z_{l}^{\prime }\right)$$

#### 3.3.3. Self-Attention Mechanism

The MSA block computes attention weights using queries Q keys, and values V matrices obtained from the input vectors. The attention matrix SA is calculated as


$$SA=Softmax\left(QK^{T}\cdot \sqrt{d_{k}}\right)\cdot V=Attention\cdot V$$

The dot product attention is scaled by $\sqrt{\left\{d_{k}\right\}}$ to accommodate the dimension of the keys. The scaled dot product attention is then passed through the SoftMax function to compute attention weights.

#### 3.3.4. Multi-Head Attention (MHA)

The MSA block combines results from multiple attention heads and applies a feed-forward layer with learnable weights $W_{0}$ to generate the final output:


$$MHA=Concat\left(SA_{1},SA_{2},SA_{3},\ldots ,SA_{h}\right)\cdot W_{0}$$

Each attention head produces an attention matrix, and the concatenated results are fed into the feed-forward layer with weights $W_{0}$. A total of 16 heads are applied.

#### 3.3.5. Output Logits

The final output from the transformer encoder, denoted as $Z_{L}$, is fed into an external linear classifier to predict class labels for image classification.

During training, the stochastic gradient is used as an optimizer, weighted categorical cross entropy is applied as a loss function, and dropout rate remains 0.2. During training, the batch is set to 32, and the learning rate remains 0.001.

## 4. Results and Discussion

For evaluating the preprocessing results, we have used two performance measures: mean squared error (MSE) as well as peak-signal-to-noise-ratio (PSNR). The MSE represents the squared cumulative error between the original and improved images. The quality of a picture improves when the MSE value is low. PSNR is used to compare the quality of two images: the original and the reconstructed picture. The higher the PSNR, the greater the quality of the reconstructed image. Similarly, for the classification performance, we have accessed accuracy as well as recall. Accuracy is a reliable assessment criterion for classification issues if the data are equally distributed, not skewed, and there is no class imbalance. In general, accuracy might produce too optimistic results, particularly on imbalanced datasets. Recall assesses the ability to discern across classes.

### 4.1. Preprocessing Results

**Figure 4:**
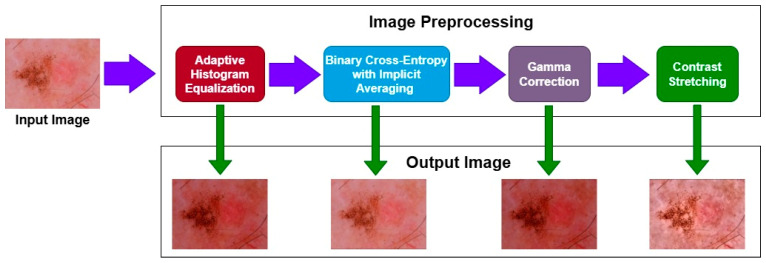
Preprocessing steps sequence.

The sequence of applying the preprocessing techniques is shown in Figure 4. Initially, we applied the AHE and obtained the resultant image. That image was then passed to the BCEI; afterwards, gamma correction was applied to the image, and finally contrast stretching was performed. The numerical result of each technique is shown in Table 2. We can see that after the image is passed through the techniques, the value of PSNR increases, and the values of MSE start decreasing, which shows that the quality of the image became better after each step.

Initially, upon applying the AHE, the value of PSNR was 0.3205, which was not very bad, but the value of MSE 362.87 was large. After applying the BCEI, the value of PSNR became better, and the MSE reduced. After applying the final contrast stretching on the image, it was evident that the value of PSNR became 0.8136, which was far better and shows that the quality of the image became much better as compared to the original one, which was also proved by the lower MSE value.

### 4.2. Classification Results

The preprocessing images were then passed through vision transformers, and the classification results in the form of accuracy and recall were collected using various train-to-test ratios and across different steps of the methodology. The proposed model was trained on 150 epochs, and the loss function was a stochastic gradient with a learning rate of 0.001. The model was trained in Jupyter Notebook 2.7 with Python 3.13.1 libraries. Tensorflow 2.18, Keras 3.0, Numpy 2.2.0, and Matplotlib 3.10.0 are the major libraries used for performing experimentations.

**Figure 5:**
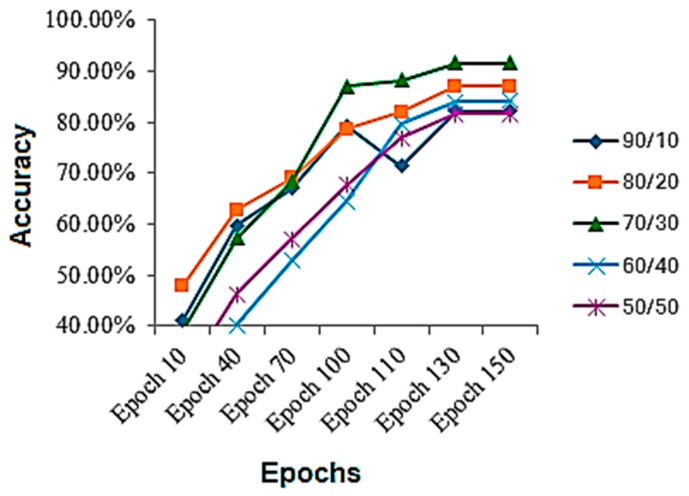
Accuracy during the training process on skin disease classification dataset.

The results were collected on various train-to-test ratios for the skin disease classification dataset, shown in Table 3. It is evident while looking at the table that the best results are achieved using a 70/30 train-to-test ratio. It can be seen that performance on a 90/10 train-to-test ratio is less, because the model gets overfitted and stop properly generalizing, and there is another reason that the model is tested on less data, which might be the reason as well. On a 50/50 ratio, the model performance also deteriorates, because the model starts underfitting and is not completely learned. Although the results are up to the marks, they are less, because the dataset is highly imbalanced, and no augmentation was performed to balance it as well as no extra steps for it were performed. If augmentation can be performed as well as other regularization techniques, if applied, the results might increase. The accuracy throughout the training process is shown in Figure 5. By analyzing the figure, we can assess that, mostly, the graph of accuracy was rising throughout the training process, but for the 70/30 ratio, the sudden jump in the accuracy was seen after the 70th epoch, after which the model generalized better afterwards.

**Figure 6:**
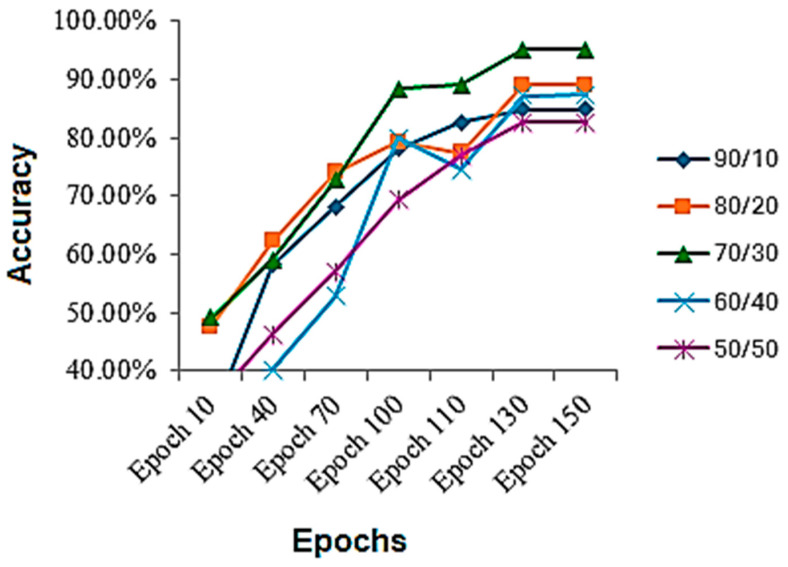
Accuracy during the training process on HAM10000 dataset.

The results are collected on various train-to-test ratios for the HAM10000 dataset, shown in Table 4. The results on the given dataset are better than the previous one. It is evident while looking at the table that, on this dataset, the best results are also achieved using a 70/30 train-to-test ratio. It can be seen that, again, the performance on a 90/10 train-to-test ratio is less, because the model gets overfitted and stops properly generalizing, and there is another reason that the model is tested on less data, which might be the reason as well. Again, on a 50/50 ratio, the model performance also deteriorates, because the model starts underfitting and is not completely learned. The accuracy throughout the training process is shown in Figure 6. By analyzing the figure, we can assess that mostly the graph of accuracy was rising throughout the training process, but for the 60/40 and 80/20, a sudden fall in the accuracies is observed in middle epochs and then raised above.

As we know, the best performance on both datasets was achieved in a 70/30 train-to-test ratio; therefore, we consider it a model. Finally, the results collected before and after preprocessing were gathered to compare the classification performance and analyze the importance of the preprocessing steps. The results are shown in Table 5; these results are collected on 70/30 train-to-test ratios. It is evident that, after the preprocessing part, both the accuracy and recall rate jumped over 8%, compared with the dataset without preprocessing, which shows the importance of the step. Moreover, we have seen that the accuracy jumped by a definite proportion for each preprocessing step. A greater jump in accuracy is observed when BCEI is applied to the dataset after AHE. Furthermore, in the last preprocessing step where we have applied contrast stretching, the accuracy jump is also significant.

**Figure 7:**
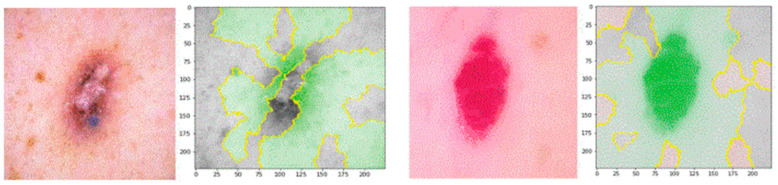
Proposed model explanations using lime.

For the explanation of the model, predictions are interpreted using the lime framework. The lime framework is used in the research as a technique to explain the model predictions for clinical interpretation [37]. Figure 7 explains the predictions of the model using lime.

Finally, we compared our proposed approach with the state-of-the art model, as shown in Table 6.

## 5. Conclusions

The research process in this study focuses on a framework for skin disease classification. The idea behind the foundation of the framework originated from the realization that the existing systems are, though efficient and precise, not very versatile, especially in terms of the number of diseases that the system can identify. This research, therefore, sought to address this gap using advanced image processing techniques with specific reference to vision transformers. Based on the presented results, it is concluded that each preprocessing technique significantly improves the classification performance. In the future, the postprocessing techniques on the classification results may be applied to evaluate the impact.

## Figures and Tables

**Table 1:** Distribution of images across skin disease image dataset.

Class	Sample Images	Number of Images
Eczema	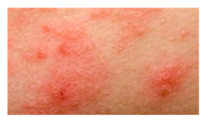	1677
Warts Molluscum and Other Viral Infections	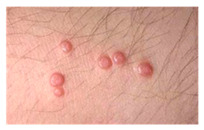	2103
Melanoma	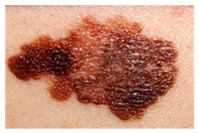	3140
Atopic Dermatitis	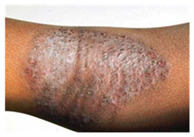	1257
Basal Cell Carcinoma	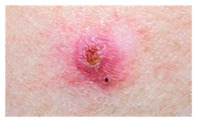	3323
Melanocytic Nevi	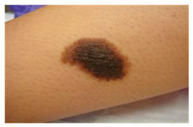	7970
Benign Keratosis-like Lesions	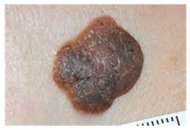	2079
Psoriasis, Lichen Planus and related diseases	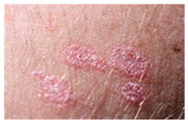	2055
Seborrheic Keratoses	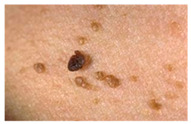	1847
Tinea Ringworm Candidiasis	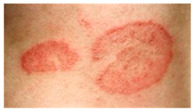	1702

**Table 2:** Numerical results of contrast enhancement.

Performance Measure	AHE	BCEI	Gamma Correction	Contrast Stretching
PSNR	0.3205	0.4987	0.7044	0.8136
MSE	362.87	281.32	199.20	110.31

**Table 3:** Classification performance on various train/test ratios for skin disease classification dataset.

Train-to-Test Ratio	Recall	Accuracy	Precision	F1-Score
90/10	81.26%	82.13%	83.44%	82.34%
80/20	85.82%	87.10%	87.29%	86.55%
70/30	89.11%	91.64%	93.01%	91.02%
60/40	84.73%	84.21%	86.33%	85.52%
50/50	80.41%	81.66%	82.55%	81.47%

**Table 4:** Classification performance on various train/test ratios for HAM10000 dataset.

Train-to-Test Ratio	Recall	Accuracy	Precision	F1-Score
90/10	82.26%	84.93%	85.12%	83.67%
80/20	86.19%	89.02%	90.23%	88.16%
70/30	94.44%	95.20%	96.87%	95.64%
60/40	84.02%	87.36%	87.96%	85.95%
50/50	80.50%	82.71%	84.01%	82.22%

**Table 5:** Classification performance comparison.

Dataset	Steps	Recall	Accuracy
Skin Disease Classification Dataset	Initial Input Dataset (Without Preprocessing)	75.18%	76.20%
	After Processing the Dataset by Applying only AHE	78.32%	79.64%
	After Processing the Dataset by Applying AHE and BCEI	84.43%	85.01%
	After Processing the Dataset by Applying AHE, BCEI, and Gamma Correction	86.71%	87.55%
	After Complete Preprocessing	89.11%	91.64%
HAM10000 Dataset	Initial Input Dataset (Without Preprocessing)	86.28%	86.98%
	After Processing the Dataset by Applying only AHE	87.33%	87.55%
	After Processing the Dataset by Applying AHE and BCEI	90.20%	91.98%
	After Processing the Dataset by Applying AHE, BCEI, and Gamma Correction	92.61%	93.04%
	After Complete Preprocessing	94.44%	95.20%

**Table 6:** Performance comparison.

Reference	Model	Accuracy
[38]	CNN	92.9%
[39]	AlexNet	93.3%
[40]	Deep CNN	90.42%
[41]	ResNet and SVM	94.5%
[42]	Inception	82.8%
[43]	Vision Transformer	93.8%
Proposed Model		95.20%

## Data Availability

The original contributions presented in the study are included in the article; further inquiries can be directed to the corresponding author.

## References

[1] Gulzar M.A., Iqbal S., Jamil A., Hameed A.A., Soleimani F. (2024). Skin Disease Detection and Classification. Intelligent Data Analytics for Bioinformatics and Biomedical Systems.

[2] Li Q., Patrick M.T., Sreeskandarajan S., Kang J., Kahlenberg J.M., Gudjonsson J.E., He Z., Tsoi L.C. (2024). Large-scale epidemiological analysis of common skin diseases to identify shared and unique comorbidities and demographic factors. Front. Immunol..

[3] Aboulmira A., Hamid H., Mohamed L. (2024). Skin Diseases Classification with Machine Learning and Deep Learning Techniques: A Systematic Review. Int. J. Adv. Comput. Sci. Appl..

[4] Pulsipher K.J., Szeto M.D., Rundle C.W., Presley C.L., Laughter M.R., Dellavalle R.P. (2021). Global Burden of Skin Disease Representation in the Literature: Bibliometric Analysis. JMIR Dermatol..

[5] Carr S., Smith C., Wernberg J. (2020). Epidemiology and Risk Factors of Melanoma. Surg. Clin..

[6] Karimkhani C., Dellavalle R.P., Coffeng L.E., Flohr C., Hay R.J., Langan S.M., Nsoesie E.O., Ferrari A.J., Erskine H.E., Silverberg J.I. (2017). Global Skin Disease Morbidity and Mortality: An Update From the Global Burden of Disease Study 2013. JAMA Dermatol..

[7] Yadav R., Aruna B. (2024). A systematic literature survey on skin disease detection and classification using machine learning and deep learning. Multimed. Tools Appl..

[8] Dip S.A., Arif K.H.I., Shuvo U.A., Khan I.A., Meng N. Equitable Skin Disease Prediction Using Transfer Learning and Domain Adaptation. Proceedings of the AAAI Symposium Series.

[9] Fuqua T. (2024). Essentials of Human Diseases and Conditions-E-Book: Essentials of Human Diseases and Conditions-E-Book.

[10] Brown M., Williams A., Chilcott R.P., Brady B., Lenn J., Evans C., Allen L., McAuley W.J., Beebeejaun M., Haslinger J. (2024). Topically applied therapies for the treatment of skin disease: Past, present, and future. Pharmacol. Rev..

[11] Jaworek A.K., Pełka K., Kozicka K., Kaleta K., Suchy W., Wójkowska-Mach J., Wojas-Pelc A. (2024). Current challenges in diagnosing and treating infectious skin diseases—A case series. Przegląd Epidemiol..

[12] Sreekala K., Rajkumar N., Sugumar R., Sagar K.D., Shobarani R., Krishnamoorthy K.P., Saini A.K., Palivela H., Yeshitla A. (2022). Skin Diseases Classification Using Hybrid AI Based Localization Approach. Comput. Intell. Neurosci..

[13] Hemanth D.J., Deperlioglu O., Kose U. (2019). An enhanced diabetic retinopathy detection and classification approach using deep convolutional neural network. Neural Comput. Appl..

[14] Zhang J., Zhong F., He K., Ji M., Li S., Li C. (2023). Recent Advancements and Perspectives in the Diagnosis of Skin Diseases Using Machine Learning and Deep Learning: A Review. Diagnostics.

[15] Rodrigues M., Ezzedine K., Hamzavi I., Pandya A.G., Harris J.E., Vitiligo Working Group (2017). New discoveries in the pathogenesis and classification of vitiligo. J. Am. Acad. Dermatol..

[16] Singh M. (2019). Cytokines: The yin and yang of vitiligo pathogenesis. Expert Rev. Clin. Immunol..

[17] Nouman Noor M., Nazir M., Khan S.A., Song O.-Y., Ashraf I. (2023). Efficient Gastrointestinal Disease Classification Using Pretrained Deep Convolutional Neural Network. Electronics.

[18] Alhajlah M., Noor M.N., Nazir M., Mahmood A., Ashraf I., Karamat T. (2023). Gastrointestinal diseases classification using deep transfer learning and features optimization. Comput. Mater. Contin.

[19] Noor M.N., Nazir M., Ashraf I., Almujally N.A., Aslam M., Fizzah Jilani S. (2023). GastroNet: A robust attention-based deep learning and cosine similarity feature selection framework for gastrointestinal disease classification from endoscopic images. CAAI Trans. Intell. Technol..

[20] Nouman Noor M., Nazir M., Khan S.A., Ashraf I., Song O.Y. (2023). Localization and classification of gastrointestinal tract disorders using explainable AI from endoscopic images. Appl. Sci..

[21] Noor M.N., Nazir M., Rehman S., Tariq J. Sketch-recognition using pre-trained model. Proceedings of the National Conference on Engineering and Computing Technology.

[22] Du-Harpur X., Watt F.M., Luscombe N.M., Lynch M.D. (2020). What is AI? Applications of artificial intelligence to dermatology. Br. J. Dermatol..

[23] Chen M., Zhou P., Wu D., Hu L., Hassan M.M., Alamri A. (2020). AI-Skin: Skin disease recognition based on self-learning and wide data collection through a closed-loop framework. Inf. Fusion.

[24] Kawahara J., Hamarneh G. (2016). Multi-Resolution-Tract CNN with Hybrid Pretrained and Skin-Lesion Trained Layers.

[25] Ismael S.A.A., Mohammed A., Hefny H. (2020). An enhanced deep learning approach for brain cancer MRI images classification using residual networks. Artif. Intell. Med..

[26] Garnavi R., Aldeen M., Celebi M.E., Varigos G., Finch S. (2021). Border detection in dermoscopy images using hybrid thresholding on optimized color channels. Comput. Med. Imaging Graph. Off. J. Comput. Med. Imaging Soc..

[27] Chieregato M., Frangiamore F., Morassi M., Baresi C., Nici S., Bassetti C., Bnà C., Galelli M. (2022). A hybrid machine learning/deep learning COVID-19 severity predictive model from CT images and clinical data. Sci. Rep..

[28] Codella N.C., Nguyen Q.B., Pankanti S., Gutman D.A., Helba B., Halpern A.C., Smith J.R. (2017). Deep learning ensembles for melanoma recognition in dermoscopy images. IEEE Xplore.

[29] Amin J., Sharif A., Gul N., Anjum M.A., Nisar M.W., Azam F., Bukhari S.A.C. (2020). Integrated design of deep features fusion for localization and classification of skin cancer. Pattern Recognit. Lett..

[30] Thomas S.M., Lefevre J.G., Baxter G., Hamilton N.A. (2021). Interpretable deep learning systems for multi-class segmentation and classification of non-melanoma skin cancer. Med. Image Anal..

[31] Al-Masni M.A., Kim D.H., Kim T.S. (2020). Multiple skin lesions diagnostics via integrated deep convolutional networks for segmentation and classification. Comput. Methods Programs Biomed..

[32] El-Khatib H., Popescu D., Ichim L. (2020). Deep Learning–Based Methods for Automatic Diagnosis of Skin Lesions. Sensors.

[33] https://www.kaggle.com/datasets/ismailpromus/skin-diseases-image-dataset.

[34] Tschandl P. (2018). The HAM10000 Dataset, a Large Collection of Multi-Source Dermatoscopic Images of Common Pigmented Skin Lesions.

[35] Ahrari A., Elsayed S., Sarker R., Essam D., Coello C.A.C. (2023). Revisiting Implicit and Explicit Averaging for Noisy Optimization. IEEE Trans. Evol. Comput..

[36] Beliakov G., Sola H.B., Sánchez T.C. (2016). A Practical Guide to Averaging Functions.

[37] Metta C., Beretta A., Guidotti R., Yin Y., Gallinari P., Rinzivillo S., Giannotti F. (2024). Advancing Dermatological Diagnostics: Interpretable AI for Enhanced Skin Lesion Classification. Diagnostics.

[38] Polat K., Kaan O.K. (2020). Detection of skin diseases from dermoscopy image using the combination of convolutional neural network and one-versus-all. J. Artif. Intell. Syst..

[39] Shanthi T., Sabeenian R.S., Anand R. (2020). Automatic diagnosis of skin diseases using convolution neural network. Microprocess. Microsyst..

[40] Kaur R., GholamHosseini H., Sinha R., Lindén M. (2022). Melanoma classification using a novel deep convolutional neural network with dermoscopic images. Sensors.

[41] Zhang L., Yang W., Chen Y. (2022). ResNet and SVM for skin disease classification on DermNet dataset with data augmentation. Appl. Soft. Comput..

[42] Yu H.Q., Reiff-Marganiec S. (2021). Targeted ensemble machine classification approach for supporting IoT enabled skin disease detection. IEEE Access.

[43] Xin C., Liu Z., Zhao K., Miao L., Ma Y., Zhu X., Zhou Q., Wang S., Li L., Yang F. (2022). An improved transformer network for skin cancer classification. Comput. Biol. Med..

